# Acarophagous Beetles Associated with *Oligonychus yothersi* (Acari: Tetranychidae) in Avocado Orchards: Life-History Traits and Responses to Infested-Plant Volatiles and Prey-Derived Odours

**DOI:** 10.3390/insects17101048

**Published:** 2026-10-12

**Authors:** Tommy Rioja, Karina B. Ruiz, Ricardo Ceballos

**Affiliations:** 1 Departamento de Recursos Ambientales, Facultad de Ciencias Agronómicas, Universidad de Tarapacá, Arica 1000000, Chile; 2 Química y Farmacia, Facultad de Ciencias de la Salud, Universidad Arturo Prat, Iquique 1110939, Chile; 3 Laboratory of Insect Chemical Ecology, Instituto de Investigaciones Agropecuarias (INIA) Quilamapu, Chillán 3800062, Chile; rceballos@inia.cl

**Keywords:** Coleoptera, fruit tree, biological parameters, olfactory responses, tri-trophic interactions, *Parastethorus histrio*, *Oligota pygmaea*, volatile organic compounds

## Abstract

The acarophagous beetles *Parastethorus histrio* (Chazeau) (Coccinellidae) and *Oligota pygmaea* (Solier) (Staphylinidae) are naturally associated with colonies of the avocado red mite, *Oligonychus yothersi* (McGregor) (Acari: Tetranychidae), in commercial avocado orchards. However, their demographics and responses to plant- and prey-derived cues remain poorly understood. Volatile profiles vary with herbivore density, plant condition, and environment, but early-autumn volatiles from mite-infested avocado shoots and their effects on these predators remain uncharacterised. We evaluated the development, survival, reproduction and fertility life-table parameters of both predators when feeding on *O. yothersi*. We also characterised volatile extracts from non-infested and mite-infested avocado shoots collected during early autumn and assessed predator responses to plant volatiles, live-prey odours and prey-derived residues in two-choice olfactometer assays. Both beetles completed their development on *O. yothersi*, with egg-to-adult survival rates of 86.6% in *P. histrio* and 90.0% in *O. pygmaea*. *Parastethorus histrio* developed more rapidly and exhibited greater female longevity, whereas *O. pygmaea* showed higher daily and lifetime fecundity. Both predators had higher net reproductive rates than the prey, but their lower intrinsic rates of increase and longer generation times indicated slower population growth per unit time. Volatile profiles differed between non-infested and mite-infested shoots, with limonene predominating in extracts from infested plants. Both predators allocated more time to extracts from mite-infested than non-infested shoots. Their responses to prey-derived cues differed: *O. pygmaea* responded positively to odours from live *O. yothersi* females but not to colony residues, whereas *P. histrio* reduced its allocation to both stimuli. These findings reveal contrasting life-history and host-location strategies in the two predators and support further evaluation of their potential as natural enemies of *O. yothersi* in avocado orchards. Field studies are required to determine whether these traits translate into predator establishment and effective suppression of mite populations.

## Simple Summary

The beetles *Parastethorus histrio* and *Oligota pygmaea* (Insecta: Coccinellidae, Staphylinidae) are found in avocado orchards infested with *Oligonychus yothersi*, yet little is known about their biology and behaviour. We investigated the biological characteristics and life-table parameters of both predatory beetles. They completed the life cycle when reared on colonies of *O. yothersi* (red mite) and showed higher net reproductive rates (*R*_0_) than the red mite. Although the intrinsic rate of increase (*r_m_*) was lower for the beetles than for the red mite, the combined population parameters suggest that both predators could still produce substantial offspring per generation over the long term once established. Furthermore, beetles of both species were attracted to herbivore-induced plant volatiles (HIPVs) emitted by avocado shoots infested with *O. yothersi*. In contrast to *O. pygmaea*, *P. histrio* displayed no positive response to odours associated with live prey or prey residues. These findings suggest that both predators may have a strong capacity to locate their prey and establish themselves within red mite colonies.

## 1. Introduction

The avocado red mite, *Oligonychus yothersi* (McGregor) (Acari: Tetranychidae), is an important foliar pest of avocado orchards in Chile [1]. By disrupting leaf tissues and extracting cellular contents, mite feeding alters plant physiological processes and causes the characteristic reddish discolouration of infested leaves [2,3,4]. Colonies generally develop along the central vein on the adaxial leaf surface and subsequently spread across the leaf as infestation levels increase [2,5]. This pest is particularly relevant in central Chile, which contains approximately 55–65% of commercial avocado orchards and where populations of *O. yothersi* increase from mid-summer to late autumn [1,6].

Two specialist acarophagous beetles, *Parastethorus histrio* (Chazeau) (Coleoptera: Coccinellidae) and *Oligota pygmaea* (Solier) (Coleoptera: Staphylinidae), are naturally associated with *O. yothersi* colonies (Figure 1A,B) in Chilean avocado orchards (Figure 1C) and prey on different developmental stages of the mite [5,7]. The species differ markedly in their life histories. *Parastethorus histrio* completes its development on mite-infested leaves, whereas late-instar larvae of *O. pygmaea* leave the foliage and pupate in dark and humid refuges within the orchard litter [8,9]. Despite their recurrent association with *O. yothersi*, the demographic performance of these predators on the avocado red mite remains insufficiently understood. In particular, fertility life-table parameters have not been reported for *P. histrio*, and available information for *O. pygmaea* is limited mainly to its development on *O. yothersi* and its demographic performance on *Oligonychus coffeae* (Nietner) (Acari: Tetranychidae) [8,9].

Development, survival, fecundity, and population growth parameters provide complementary information about prey suitability for a predator and its potential for population establishment. Parameters such as the net reproductive rate (*R*_0_), intrinsic rate of increase (*r_m_*), finite rate of increase (*λ*), mean generation time (*T*) and doubling time (*DT*) can be used to compare lifetime reproductive output with the temporal rate of population growth [10,11,12]. This distinction matters when comparing predators with rapidly reproducing prey: high lifetime fecundity does not necessarily mean predator populations can increase as quickly as the pest population. Moreover, demographic performance alone cannot explain whether a predator can locate spatially dispersed prey colonies. Evaluating predator potential therefore requires consideration of both population traits and prey-searching behaviour.

Herbivore-induced plant volatiles (HIPVs) can provide natural enemies with reliable information about herbivores’ presence and location. Feeding by phytophagous mites activates plant defence responses and modifies the composition and abundance of emitted volatile organic compounds, including green leaf volatiles, terpenoids, jasmonates and aromatic compounds [13,14,15,16,17]. These volatile blends may mediate tritrophic interactions by attracting predators to herbivore-infested plants [18,19,20]. Previous studies have shown that *P. histrio* and *O. pygmaea* respond to volatiles from *O. yothersi*-infested avocado plants and to selected synthetic compounds, including limonene, ocimene, farnesene and methyl salicylate [5]. However, volatile profiles can vary with herbivore density, plant condition and environmental context, and the composition of volatiles emitted by mite-infested avocado shoots during early autumn has not been characterised in relation to the behavioural responses of these predators.

Predators may also use cues emitted directly by their prey or deposited within prey colonies, including faeces, silk and exuviae. The relative importance of plant- and prey-derived cues appears to vary among acarophagous natural enemies. For example, odours from *O. yothersi* were not attractive to the predatory mite *Galendromus occidentalis* (Nesbitt) (Acari: Phytoseiidae) in Y-tube assays, whereas the acarophagous beetle *Stethorus gilvifrons* (Mulsant) (Coleoptera: Coccinellidae) responded to kairomones associated with *Tetranychus urticae* Koch (Acari: Tetranychidae) [21,22]. Whether *P. histrio* and *O. pygmaea* respond similarly to prey-derived odours and colony residues remains unknown. Comparing their responses to plant- and prey-associated cues could therefore reveal differences in the mechanisms these predators use to locate *O. yothersi* colonies.

This study aimed to determine the developmental, reproductive and fertility life-table parameters of *P. histrio* and *O. pygmaea* when feeding on *O. yothersi*, characterise the volatile profiles of non-infested and mite-infested avocado shoots during early autumn, and evaluate predator responses to plant volatiles, live-prey odours and prey-associated residues. We hypothesised that (i) *O. yothersi* would support the complete development and reproduction of both beetle species; (ii) both predators would preferentially respond to volatile extracts from mite-infested rather than non-infested avocado shoots; and (iii) their responses to prey-derived odours and colony residues would differ between species. By combining these complementary measurements, we sought to establish a baseline for understanding the demographic performance and host-location behaviour of these acarophagous beetles and for guiding their future evaluation as biological control agents in avocado orchards.

## 2. Materials and Methods

### 2.1. Rearing of Oligonychus yothersi

*Oligonychus yothersi* was established from individuals collected in March 2023 from Hass avocado plants in La Cruz, central Chile (32°49′ S, 71°17′ W). The colonies were subsequently maintained on ‘Hass’ avocado leaf discs (8.5 cm in diameter) placed with the abaxial surface in contact with moist cotton inside plastic Petri dishes (9 cm in diameter and 1.5 cm in height). Each dish contained an opening in its base covered with muslin and was placed on a moist sponge inside a plastic tray to prevent dehydration of the leaf material and escape of the mites (Figure 2A). Leaf discs were replaced periodically to maintain suitable feeding conditions and colony quality.

For two-choice olfactory assays involving the previous feeding of beetle predators, *Tetranychus urticae* (Koch) (Acari: Tetranychidae) was reared on castor bean, *Ricinus communis* L., plants grown in 1 L pots. Mite-infested plants were maintained in muslin cages (30 × 50 × 30 cm) under laboratory conditions at 23 ± 3 °C, 60 ± 10% RH, and a 16/8 h day/night photoperiod.

### 2.2. Collection and Rearing of Predatory Beetles

Adults of *Parastethorus histrio* (Chazeau) (Coleoptera: Coccinellidae) and *Oligota pygmaea* (Solier) (Coleoptera: Staphylinidae) were taxonomically identified and collected from *O. yothersi*-infested avocado trees located in La Cruz (central Chile, 32°49′ S, 71°17′ W). The beetles were transported to the laboratory in ventilated containers containing avocado leaves naturally infested with *O. yothersi*. Laboratory colonies of *P. histrio* and *O. pygmaea* were established from 100 field-collected adults each. All experiments used individuals from the first laboratory generation (F1).

For colony establishment and egg production, thirty adults of each predator species were placed on *O. yothersi*-infested avocado leaf discs inside Petri dishes for 24 h. Adults were subsequently transferred to new infested leaf discs. Each dish was covered with a second Petri dish containing a muslin-covered opening to prevent beetle escape and excessive accumulation of humidity (Figure 2A–C). To obtain newly emerged *P. histrio* adults, pupae attached to avocado leaf discs were transferred to transparent plastic containers (11 cm in diameter and 14 cm in height) containing a layer of gypsum plaster at the bottom. For *O. pygmaea*, late third-instar larvae were transferred individually to similar containers containing a 5 cm deep gypsum plaster layer with irregular cavities that provided shelters for pupation [9]. The plaster was moistened every three days, or whenever a colour change indicated moisture loss. Containers were covered with black cloth to provide the dark conditions required for pupation (Figure 2D–I). Colonies were maintained in an incubator at 25 ± 0.7 °C, 60 ± 5% RH and a 16/8 h day/night photoperiod.

### 2.3. Development and Survival

To evaluate the development of *P. histrio*, adult females from different maternal lines were allowed to oviposit for 2 h on avocado leaf discs containing colonies of *O. yothersi*. After oviposition, we removed the females and carefully transferred the eggs to new infested leaf discs. One egg was placed in each Petri dish, and each individual constituted an independent experimental unit (n = 30). After hatching, larval instars were identified based on the presence of exuviae, which were removed using a fine brush after each moult. Embryonic development, duration of each immature stage, survival and adult longevity were recorded every 24 h using a stereomicroscope. Infested leaf discs were replaced every ten days or earlier when deterioration of the leaf material or depletion of prey was observed. The same procedure was followed for *O. pygmaea*. However, late third-instar larvae were transferred individually to transparent plastic containers containing a 5 cm deep gypsum plaster base with irregular cavities. The containers were covered with muslin and black cloth to provide suitable humidity and darkness for pupation. Following adult emergence, beetles were individually transferred to new avocado leaf discs containing *O. yothersi* colonies. We evaluated prey development using eggs of known age obtained from different *O. yothersi* females. Females were allowed to oviposit on avocado leaf discs for 2 h, after which adults and excess eggs were removed, leaving one egg per experimental unit (n = 30). Developmental stages were identified from the exuviae produced at each moult. All experimental units were maintained at 25 ± 0.7 °C, 60 ± 5% RH and a 16/8 h day/night photoperiod. Immature development and survival were assessed using cohorts initiated with 30 eggs, while longevity and reproductive parameters were evaluated using a separate group of 30 adult females. Thus, the females used for reproductive measurements were not derived from the cohorts used to estimate immature survival. Observations continued until each adult died. Prey was provided ad libitum throughout the experiment, with additional mites added as needed to prevent depletion.

### 2.4. Reproduction and Fertility Life-Table Parameters

Newly emerged *P. histrio* females from different maternal lines were individually transferred to avocado leaf discs containing *O. yothersi* colonies. Each leaf disc was maintained inside a plastic Petri dish and represented one experimental unit. Two males were introduced into each dish for 24 h every seven days to ensure mating. Eggs laid by each female were counted and removed every 24 h. Each female constituted one biological replicate (n = 30), and observations continued until female death, and adult female longevity was recorded for each individual. The same procedure was followed for *O. pygmaea*. To determine offspring sex ratio, eggs produced by ten randomly selected females were maintained individually until adult emergence. Late third-instar larvae were transferred to individual containers containing a gypsum plaster base with irregular cavities and maintained under dark conditions until pupation and adult emergence. For *O. yothersi*, newly emerged females were individually transferred to avocado leaf discs and continuously maintained with two males throughout the reproductive assessment [1]. Eggs were counted and removed every 24 h. Eggs produced by ten randomly selected females were transferred to new leaf discs and maintained until adulthood to determine offspring sex ratio. All experimental units were maintained at 25 ± 0.7 °C, 60 ± 5% RH and a 16/8 h day/night photoperiod. The pre-oviposition, oviposition and post-oviposition periods, total fecundity, mean daily fecundity and female longevity were recorded. Female age-specific survival (*l_x_*) and fecundity (*m_x_*) were used to construct female age-specific fertility life tables.

Life-table parameters of *O. yothersi* and its predators, *P. histrio* and *O. pygmaea*, were compared to assess their population growth potential within the same predator–prey system. Demographic parameters, particularly the intrinsic rate of increase and generation time, are commonly used to evaluate the relative population growth of arthropod pests and their natural enemies. Accordingly, this comparison examined whether differences in these parameters could help explain the demographic relationships between *O. yothersi* and its two predators under the experimental conditions, rather than imply demographic equivalence among the species.

### 2.5. Plant Material and Mite Infestation

Two-year-old ‘Hass’ avocado plants grafted onto ‘Mexicola’ rootstock were grown individually in 7 L pots containing organic soil. Plants were maintained under semi-field conditions in two physically separated anti-aphid-screened greenhouses and were irrigated and fertilised with Ultrasol^®^ Multipurpose 18-18-18 (Soquimich, Santiago, Chile) according to crop requirements. We used twelve plants: six non-infested control plants and six plants infested with *Oligonychus yothersi*. We maintained the two treatments in separate greenhouses to minimise volatile-mediated signalling between control and infested plants. For the infestation treatment, we obtained avocado leaf discs containing sixty *O. yothersi* individuals from the laboratory colony. We removed excess mites under a stereomicroscope to standardise the initial density. One infested disc was attached with a clip to the abaxial surface of each leaf on a selected mid-canopy shoot. Each selected shoot bore four to six leaves and therefore received four to six infested leaf discs. A sticky barrier (Stikem Special, SEABRIGHT Laboratories, Emeryville, CA, USA) was applied at the base of each shoot to prevent mites from dispersing to other parts of the plant. Control shoots underwent the same manipulation using non-infested avocado leaf discs, clips, and sticky barriers. After 48 h, we removed all leaf discs and clips from both control and infested shoots. Before VOC collection, the number of adult mites per leaf on each infested shoot was verified using a 10× handheld magnifying glass [5]. Volatile collection was conducted after three days of exposure to the corresponding treatment.

### 2.6. Collection and Chemical Analysis of Avocado Volatiles

Volatile organic compounds were collected simultaneously from six non-infested and six *O. yothersi*-infested avocado plants after three days of treatment. One mid-canopy shoot from each plant was enclosed in an individual transparent 1 L food-grade oven bag. Each plant constituted an independent sampling unit within its corresponding greenhouse. Dynamic headspace sampling was conducted by introducing charcoal-filtered air into each bag (ALTEF gas sample bag, RESTEK, Bellefonte, PA, USA) at 1000 mL min^−1^. Air was simultaneously withdrawn at 900 mL min^−1^ through a glass trap containing 100 mg of Porapak Q. Volatiles were collected continuously for 24 h under semi-field conditions during early autumn. The environmental conditions were monitored using a data logger and information from the local meteorological station ‘La Cruz’ (https://agrometeorologia.cl/). Following collection, each Porapak Q trap was eluted separately with 1 mL of chromatographic-grade hexane. We kept the six extracts from each treatment as independent biological samples and did not pool them. Extracts were stored in amber glass vials at −80 °C until chemical and behavioural analyses. Before and after each reuse, the traps were rinsed with 1 mL of redistilled diethyl ether (Merck, Darmstadt, Germany) and conditioned under nitrogen flow at 150 °C for 2 h. Volatile extracts were analysed using a GCMS-QP2010 Ultra gas chromatograph–mass spectrometer (Shimadzu, Kyoto, Japan) equipped with an RTx-5 capillary column. One microlitre of each extract was injected in splitless mode. The oven temperature was maintained at 40 °C for 2 min, then increased to 225 °C at 5 °C min^−1^. Helium was used as the carrier gas at 1 mL min^−1^. Mass spectra were acquired in electron-impact ionisation mode at 70 eV over an *m*/*z* range of 50–500, with the ion source maintained at 230 °C. Compounds were tentatively identified by comparing their mass spectra with the NIST library. Blank collections were analysed using the same procedure, and compounds detected in the blanks were excluded from the avocado volatile profiles. Only matches ≥ 90% were retained. Where available, retention times were also used to evaluate compound identities. Methyl jasmonate at 10 µg mL^−1^ was used as the analytical standard. Because compound-specific calibration curves were not generated, volatile abundances were estimated semiquantitatively as methyl jasmonate equivalents rather than as absolute concentrations. These values should therefore be considered relative estimates, particularly for compounds outside the standard concentration range, such as limonene. Limonene identity was confirmed by comparison with an authentic (R)-(+)-limonene analytical standard (Sigma-Aldrich, St. Louis, MO, USA).

### 2.7. Two-Choice Olfactory Assays

Olfactory responses of *P. histrio* and *O. pygmaea* females were evaluated using a glass Y-tube olfactometer with two 8 cm arms, an internal diameter of 1 cm and an angle of 60°. The olfactometer was divided into a stimulus arm, a control arm and a basal decision zone. The following two-choice comparisons were evaluated:

1. air versus air;
2. hexane versus air;
3. non-infested avocado extract versus hexane;
4. mite-infested avocado extract versus hexane;
5. mite-infested versus non-infested avocado extract;
6. live *O. yothersi* odour versus air;
7. *O. yothersi*-associated residues versus air.

For assays involving plant volatiles, we applied 50 µL of extract or hexane to a strip of Whatman No. 1 filter paper. The solvent was allowed to evaporate before the filter paper was introduced into the glass odour chamber. We placed it inside a glass odour chamber connected to the corresponding olfactometer arm. Charcoal-filtered and humidified air was supplied through both arms and withdrawn through the basal zone at 300 mL min^−1^. For each plant treatment, extracts from six biological replicates were combined in equal proportions to create a pooled extract, which served as the odour source for the corresponding olfactometer comparison. The six plant extracts were therefore not tested separately in the behavioural assays. Each two-choice comparison included 20 predator females, with each individual used only once. In the air-versus-air and hexane-versus-air controls, 10 females of each predator species were included.

For live-prey odour assays, 200 adult *O. yothersi* females were confined for 2 h inside the glass chamber connected to the stimulus arm. Mites were tested in the absence of avocado leaves to isolate mite-derived odours from plant-derived volatile cues [21]. In contrast, the control-arm chamber remained empty. For residue assays, faeces, silk and exuviae were collected from *O. yothersi* colonies and transferred to the stimulus chamber. A single predator female was introduced into the basal zone and acclimatised for 5 min before each trial. We recorded time spent in the stimulus arm, control arm, and decision zone for 10 min. Twenty-four hours before the assays, predator females were provided with water but deprived of prey. Four-day-old mated females of *P. histrio* and *O. pygmaea* obtained from laboratory colonies were used in the olfactory assays. Before testing, females were maintained on *T. urticae* to standardise their previous prey experience and prevent prior exposure to *O. yothersi*. The olfactometer was rotated 90° clockwise between consecutive trials to alternate the spatial positions of the stimulus and control arms and reduce directional responses associated with external environmental cues. A clean Y-tube was used for each trial, and the odour chambers and connecting components were cleaned between replicates. Assays were conducted between 11:00 and 16:00 h at 25 ± 2 °C and 65 ± 5% RH.

### 2.8. Statistical Analysis

#### 2.8.1. Development and Reproductive Parameters

Developmental duration and reproductive variables were examined for normality and homogeneity of variance using model residuals. Comparisons between the two predator species were performed using Student’s *t*-test or Welch’s *t*-test when variances were unequal. Variables including the two predators and their prey were analysed using one-way ANOVA followed by Tukey’s significant difference test. Data were transformed as *ln*(x + 1) when required to meet model assumptions. Results are presented on the original measurement scale as means ± SD. Statistical analyses were performed using R software (Version 4.5.3, R Core Team [23]).

#### 2.8.2. Fertility Life-Table Parameters

Female age-specific survival and fecundity were used to calculate:

(1) $$R_{0}=\;\sum l_{x}m_{x}$$

(2) $${\sum e}^{-r(x+1)}(l_{x}m_{x})=1$$

(3) $$\lambda =e^{r_{m}}$$

(4) $$T=(lnR_{0})/r_{m}$$

(5) $$DT=(ln2)/r_{m}$$

*R*_0_ represents the number of female offspring produced by a female over one generation or throughout her lifetime, considering female age-specific survival (*l_x_*) and fecundity (*m_x_*) (1); *r_m_* is the intrinsic rate of increase, corresponding to the maximum exponential growth rate of the population and calculated using (2), expressed as females female^−1^ day^−1^; *λ* the finite rate of increase (3), defined as the population multiplication factor per female per unit time; *T* is the mean generation time (4), representing the interval between successive generations; and the doubling time [*DT*, (5)], defined as the time required for the population to double in size [10]. We used jackknife resampling to obtain pseudovalues and variance estimates for each parameter using the *lifetable.r* procedure described by Maia et al. [24]. Parameters were compared among species using an overall test followed by Tukey-adjusted pairwise comparisons.

#### 2.8.3. Volatile Emissions

Volatile abundance was log-transformed as *ln*(x + 1) when necessary. Because we sampled control and infested plants simultaneously and treated them as independent experimental units, we used independent-sample tests rather than paired tests. We applied a false-discovery-rate adjustment to account for multiple comparisons across compounds.

#### 2.8.4. Olfactory Responses

For behavioural assays involving plant volatiles, the six independent extracts obtained from each plant treatment were combined in equal proportions to generate a single pooled extract per treatment, as described in Section 2.7. For each female, the time spent in the stimulus, control and decision zones was expressed as a proportion of the 600 s observation period. We used non-parametric bootstrap resampling with 10,000 iterations to estimate mean differences and 95% confidence intervals. Air–air assays were used to characterise basal spatial allocation, whereas hexane–air assays were used to quantify the effect of the solvent. We then compared experimental conditions following Ceballos et al. [21]. A response was considered directional when the 95% confidence interval for the stimulus–control contrast did not include zero.

## 3. Results

### 3.1. Development, Survival and Longevity of Parastethorus histrio and Oligota pygmaea

Both predator species completed their development when provided with colonies of *Oligonychus yothersi* on avocado leaf discs. *Parastethorus histrio* developed through four larval instars, whereas *Oligota pygmaea* developed through three larval instars followed by a comparatively prolonged pupal stage. Embryonic development was longer in *P. histrio* than in *O. pygmaea* (5.1 ± 0.4 and 4.0 ± 0.7 d, respectively; Table 1). However, the pupal stage was substantially shorter in *P. histrio* than in *O. pygmaea* (4.5 ± 0.5 and 13.0 ± 1.1 d, respectively). Consequently, total development from egg to adult was shorter in *P. histrio* (19.6 ± 1.2 d) than in *O. pygmaea* (24.4 ± 1.7 d). Survival from egg to adult was 86.6% for *P. histrio* and 90.0% for *O. pygmaea* (Table 1).

Under the same temperature and humidity conditions, *O. yothersi* completed its development from egg to adult in 8.80 ± 0.42 d, with 93.3% survival to adulthood (Table 2). Thus, the prey completed its immature development considerably earlier and had a shorter adult lifespan than either predator species.

Females of both predator species deposited eggs among mite colonies and covered them with exuviae and dead bodies of *O. yothersi* (Figure 3 and Figure 4). *Parastethorus histrio* completed its immature development on the avocado leaf surface. In contrast, late third-instar larvae of *O. pygmaea* moved into cavities in the gypsum substrate, where pupation and adult emergence occurred under dark and humid conditions.

### 3.2. Reproductive and Fertility Life-Table Parameters

The pre-oviposition period differed among the two predators and their prey (*F* = 176.88, *p* < 0.001). It was longest in *P. histrio* (3.5 ± 0.5 d), intermediate in *O. pygmaea* (2.4 ± 0.5 d) and shortest in *O. yothersi* (1.1 ± 0.4 d; Table 3). The oviposition period also differed among species (*F* = 889.75, *p* < 0.001). *Parastethorus histrio* showed the longest oviposition period, followed by *O. pygmaea* and *O. yothersi*. The post-oviposition period was longer in both predators than in the prey, but did not differ between the two predator species. Total fecundity was highest in *O. pygmaea*, which produced 441.6 ± 51.5 eggs female^−1^, followed by *P. histrio* with 351.8 ± 41.1 eggs female^−1^ and *O. yothersi* with 68.1 ± 16.1 eggs female^−1^ (*F* = 743.71, *p* < 0.001; Table 3).

Mean daily fecundity was also highest in *O. pygmaea* (7.0 ± 0.4 eggs female^−1^ d^−1^), whereas no significant difference was detected between *P. histrio* and *O. yothersi* (4.4 ± 0.2 and 4.6 ± 0.6 eggs female^−1^ d^−1^, respectively). Among the offspring obtained from the ten selected families, females represented 53.7% of emerged *P. histrio*, 56.0% of emerged *O. pygmaea* and 76.0% of emerged *O. yothersi* adults. Age-specific survival and fecundity differed markedly among species (Figure 5).

Survival of *P. histrio* females remained above 0.80 until approximately 98 d of age, whereas survival of *O. pygmaea* remained above 0.80 until approximately 90 d. In contrast, *O. yothersi* survival declined rapidly after approximately 12 d. Egg production in both predator species extended over a longer period than in *O. yothersi*, particularly in *P. histrio*, which continued ovipositing until approximately 120 d of age. Net reproductive rate differed among species (*F* = 604.60, *p* < 0.001). *Oligota pygmaea* exhibited the highest *R*_0_, with 218.63 ± 25.49 daughters female^−1^ generation^−1^, followed by *P. histrio* with 160.35 ± 18.74 and *O. yothersi* with 47.52 ± 11.21 (Table 4). In contrast, the intrinsic rate of increase was highest in *O. yothersi* (*r_m_* = 0.245 ± 0.016 d^−1^) and was approximately twice the values recorded for *P. histrio* and *O. pygmaea* (*F* = 1250.46, *p* < 0.001). The two predators did not differ in *r_m_*. The finite rate of increase showed the same pattern: *λ* was higher in *O. yothersi* than in either predator, with no significant difference between *P. histrio* and *O. pygmaea*. Mean generation time was shortest in *O. yothersi* (15.66 ± 0.97 d), followed by *P. histrio* (40.06 ± 2.96 d) and *O. pygmaea* (41.54 ± 1.51 d; *F* = 1583.75, *p* < 0.001). Similarly, the prey exhibited a shorter doubling time than either predator (*F* = 1481.56, *p* < 0.001), whereas doubling time did not differ between the two predator species. Overall, both predators produced more offspring per generation than *O. yothersi*. Still, their lower *r_m_* and *λ*, together with their longer generation and doubling times, indicated slower population growth per unit time.

### 3.3. Volatile Profiles of Non-Infested and Mite-Infested Avocado Shoots

Volatile profiles differed between non-infested avocado shoots and shoots exposed to *O. yothersi* for three days (Table 5). Across both treatments, the detected compounds included green leaf volatiles, alkanes and terpenoids. Three green leaf volatiles—3-hexanone, 2-hexanone and 3-hexanol—were detected in extracts from both control and mite-infested shoots, with no statistically significant differences between treatments. Most alkanes were also present under both conditions, although 4,7-dimethylundecane was detected only in extracts from non-infested shoots. The monoterpenes α-pinene, β-pinene and limonene were detected in both treatments. α-Pinene abundance was lower in extracts from mite-infested shoots than in controls, whereas limonene was approximately 29-fold more abundant in extracts from infested shoots (*p* < 0.05). Myrcene, β-ocimene, α-farnesene and geraniol, together with methyl salicylate, were detected only in extracts obtained from mite-infested avocado shoots. Conversely, 3-carene was detected only in extracts from non-infested shoots. Limonene was the predominant compound in the volatile extracts from mite-infested shoots, accounting for approximately 86% of the total detected volatile abundance.

### 3.4. Olfactory Responses to Avocado-Shoot Volatiles and Prey-Associated Odours

#### 3.4.1. Baseline and Solvent Controls

In air–air control assays, females of both predator species distributed their time among the two arms and the decision zone without a pronounced directional bias (Figure 6). When hexane was presented against clean air, both species spent less time in the hexane arm than expected from their corresponding air–air baseline. In *P. histrio*, the proportion of time allocated to the hexane arm decreased by 0.073 (95% CI: −0.128 to −0.019), accompanied by an increase in time spent in the decision zone (Supplementary Data S1). A similar response was observed in *O. pygmaea*, for which stimulus-arm allocation decreased by 0.072 (95% CI: −0.128 to −0.022). These results indicate that hexane moderately affected the spatial distribution of both predator species; therefore, this effect was taken into account when interpreting subsequent comparisons involving the solvent control.

#### 3.4.2. Responses to Avocado-Shoot Extracts

Neither predator showed increased allocation to the stimulus arm when extracts from non-infested avocado shoots were presented against hexane. Relative to the hexane–air reference, the estimated change was −0.021 for *P. histrio* (95% CI: −0.093 to 0.058) and −0.039 for *O. pygmaea* (95% CI: −0.111 to 0.031). In contrast, extracts from mite-infested shoots increased stimulus-arm occupancy in both predators relative to the hexane–air reference. The estimated change was +0.315 for *P. histrio* (95% CI: 0.269–0.368) and +0.361 for *O. pygmaea* (95% CI: 0.312–0.416). When extracts from mite-infested and non-infested shoots were presented simultaneously, both predator species spent more time in the arm containing the mite-infested shoot extract (Figure 7A,B) (Supplementary Data S1). The estimated increase in stimulus-arm allocation was +0.337 for *P. histrio* (95% CI: 0.229–0.426) and +0.355 for *O. pygmaea* (95% CI: 0.306–0.400).

#### 3.4.3. Responses to Live-Prey Odours and Prey-Associated Residues

The two predator species differed in their responses to odours from live *O. yothersi* females. *Parastethorus histrio* spent less time in the arm containing prey odours than expected from the air–air reference (Δ = −0.198; 95% CI: −0.244 to −0.151) (Figure 7A) (Supplementary Data S1). By contrast, *O. pygmaea* increased its allocation to the arm containing live-prey odours (Δ = +0.305; 95% CI: 0.186–0.402) (Figure 7B). A similar negative response was observed in *P. histrio* when the stimulus consisted of faeces, silk and exuviae collected from *O. yothersi* colonies (Δ = −0.222; 95% CI: −0.272 to −0.171). *Oligota pygmaea* showed no clear directional response to prey-associated residues (Δ = +0.045; 95% CI: −0.093 to 0.177). Thus, both beetle species responded positively to volatile extracts from mite-infested avocado shoots. In contrast, their responses to cues derived directly from the prey differed: *O. pygmaea* responded positively to live-prey odours, while *P. histrio* did not (Figure 7A,B).

## 4. Discussion

This study reveals complementary life-history and host-location strategies in *P. histrio* and *O. pygmaea*. Although both beetles developed and reproduced on *O. yothersi*, their demographic profiles differed in developmental duration, longevity and reproductive output. Both species discriminated against volatile extracts from mite-infested and non-infested avocado shoots, but differed in their responses to cues derived directly from the prey.

### 4.1. Oligonychus yothersi Supports Contrasting Life-History Strategies in Two Acarophagous Beetles

Both *P. histrio* and *O. pygmaea* completed their development with high immature survival when fed on *O. yothersi*, confirming that the avocado red mite can sustain the development of both predators. However, the two beetles exhibited contrasting life-history patterns. *Parastethorus histrio* developed more rapidly, completed its entire immature development on the leaf surface and showed greater adult longevity, whereas *O. pygmaea* had a prolonged pupal stage and greater daily and lifetime fecundity.

The developmental duration of *P. histrio* was within the range reported for several acarophagous coccinellids in the tribe Stethorini under comparable temperatures. Development from egg to adult requires approximately 19 d in *Stethorus gilvifrons* feeding on *Oligonychus coffeae*, whereas shorter periods have been reported when the same predator is provided with *Tetranychus urticae* or other mite–host plant combinations [25,26,27,28]. Similar developmental periods have also been reported for *Stethorus punctillum* Weise and *Stethorus punctum picipes* Casey (Coleoptera: Coccinellidae) feeding on tetranychid mites [29,30]. Variation among studies likely reflects the combined effects of predator identity, prey species, host plant and temperature [2,11,31].

The longer immature development of *O. pygmaea* largely resulted from its prolonged pupal stage. A similar developmental duration was reported for this species feeding on *O. coffeae* on tea plants, as well as in previous observations of *O. pygmaea* feeding on *O. yothersi* [8,9]. Unlike *P. histrio*, which remained on the avocado leaf throughout immature development, late-instar larvae of *O. pygmaea* required dark and humid cavities to pupate successfully. This behaviour agrees with observations of pupation within the litter layer of fruit orchards and suggests that the availability of protected microsites may influence the persistence of *O. pygmaea* populations [8,9]. The ecological importance of orchard litter for this species nevertheless remains to be tested experimentally.

The reproductive traits of the two predators revealed a second life-history contrast. *Oligota pygmaea* exhibited greater daily and total fecundity, whereas *P. histrio* combined lower daily egg production with a longer oviposition period and greater adult longevity. The fecundity of *P. histrio* was higher than values reported for several *Stethorus* species feeding on tetranychid mites, while that of *O. pygmaea* was comparable to or slightly greater than previously recorded when this species was fed *O. coffeae* [8,25,26,28,32,33]. However, make direct comparisons cautiously because reproductive performance is strongly affected by prey identity and density, host-plant quality, and temperature [2,11,31].

Females of both species deposited their eggs within mite colonies and covered them with exuviae and dead mite bodies. Although this behaviour may provide physical protection, we did not test its function experimentally. Overall, the two predators achieved similarly high immature survival through different strategies: prolonged adult persistence in *P. histrio* and greater reproductive output in *O. pygmaea*. Whether these differences influence their persistence under fluctuating prey availability remains to be determined.

### 4.2. High Lifetime Reproductive Output Does Not Offset Slower Population Growth

The fertility life tables revealed a clear distinction between reproductive output per generation and population growth per unit time. Both predators showed higher net reproductive rates than *O. yothersi*, reflecting their prolonged survival and extended oviposition periods. The *R*_0_ obtained for *O. pygmaea* was comparable to that reported when this species was reared on *O. coffeae*, whereas the value for *P. histrio* was within the broad range reported for acarophagous Stethorini under favourable laboratory conditions [8,11,25,31,32,34].

Despite their greater *R*_0_, both predators exhibited lower intrinsic and finite rates of increase and longer generation and doubling times than the prey. Because *r_m_* incorporates not only reproductive output but also the timing of reproduction, these results indicate that *O. yothersi* can increase more rapidly than either predator, even though individual predator females produce more daughters over their lifetimes [10,35]. The mite’s rapid development and early reproduction therefore compensate for its shorter reproductive period and lower lifetime fecundity.

The similar *r_m_*, *λ* and *DT* values of *P. histrio* and *O. pygmaea* indicate broadly comparable potential rates of population increase despite their contrasting life-history traits. In *O. pygmaea*, greater fecundity was partly offset by its longer generation time. In contrast, *P. histrio*’s lower daily fecundity was accompanied by faster immature development and greater female longevity. This illustrates why demographic parameters should be interpreted collectively rather than using fecundity, *R*_0_ or *r_m_* as isolated indicators of predator performance [11,21,25,35].

The life tables therefore establish that both beetles can maintain positive population growth when continuously supplied with *O. yothersi*. Still, they do not demonstrate that predator populations can numerically track or suppress the more rapidly increasing prey. Such an assessment also requires information on prey consumption, functional and numerical responses, prey-stage preference and performance across different prey densities [2,11].

### 4.3. Infested-Plant Volatiles Provide Shared Cues, Whereas Prey Odours Reveal Species-Specific Responses

Volatile profiles differed between non-infested avocado shoots and shoots exposed to *O. yothersi* for three days. Extracts from mite-infested shoots contained myrcene, β-ocimene, α-farnesene, geraniol and methyl salicylate, which were not detected in control extracts, and showed substantially greater limonene abundance. These compounds belong to volatile groups frequently associated with plant responses to herbivory and with attraction to natural enemies [13,14,15,16,17,36,37,38,39].

The composition of these extracts is consistent with previous behavioural responses of *P. histrio* and *O. pygmaea* to synthetic compounds, including limonene, ocimene, pinene, farnesene and methyl salicylate [5]. Nevertheless, the present experiment evaluated complete volatile blends rather than individual constituents. Therefore, it is not possible to determine whether predator responses were driven by limonene, compounds detected exclusively in infested shoots, changes in their relative proportions, or interactions among several components of the blend. Responses to extracts from mite-infested avocado shoots cannot be attributed to a specific component of the volatile stimulus because infestation altered both volatile composition and emission levels. Limonene dominated the profile and was more abundant in infested shoots. Therefore, the assays cannot resolve whether predator responses were driven by blend composition, volatile concentration, limonene abundance, or a combination of these factors. Concentration-matched extracts, dose–response assays, and tests with individual compounds are needed to disentangle their relative contributions.

Both beetle species allocated more time to arms containing extracts from mite-infested shoots than to those containing extracts from non-infested shoots. Previous studies similarly reported attraction of *P. histrio* and *O. pygmaea* to volatiles from *O. yothersi*-infested avocado plants during summer and late autumn [5,40]. These findings extend these observations to extracts collected in early autumn and at lower mite densities, suggesting that both predators can detect volatile differences associated with mite-infested avocado shoots across different periods of the pest’s seasonal occurrence.

The solvent-control assays showed that hexane was not behaviourally neutral. Both predators spent less time in the hexane arm and showed higher occupancy of the decision zone. Therefore, we needed to account for this effect when interpreting comparisons between plant extracts and solvent controls. In this context, comparing infested and non-infested shoot extracts provides the most informative behavioural evidence because both arms contained plant-derived samples with an equivalent solvent background.

The predators differed in their responses to cues derived directly from *O. yothersi*. *Oligota pygmaea* increased the time spent in the arm containing odours from live mite females but showed no directional response to prey-associated residues. By contrast, *P. histrio* reduced its allocation to arms containing both live-prey odours and colony residues. Its response should therefore be interpreted as a reduced response to the stimulus under the assay conditions, rather than as a simple absence of attraction.

These contrasting responses suggest that the two predators may use different combinations of olfactory information to locate prey habitats. *Oligota pygmaea* responded to both infested-plant extracts and live-prey odours, whereas the positive response of *P. histrio* was restricted to cues associated with infested shoots. A similar dependence on plant- and prey-derived cues has been reported in other acarophagous natural enemies, although the relative importance of these signals varies among predator and prey species [21,22]. Because we did not quantify the concentration and chemical composition of prey-derived stimuli, confirmation across different prey densities and stimulus concentrations is needed before these differences can be regarded as distinct searching strategies.

### 4.4. Demographic and Olfactory Traits Support Further Evaluation for Biological Control

The combination of demographic performance and olfactory discrimination observed in *P. histrio* and *O. pygmaea* provides a biological basis for their continued evaluation as natural enemies of *O. yothersi*. Both predators completed their development and maintained positive population growth on the avocado red mite. At the same time, their responses to volatile extracts from infested shoots indicate they can recognise cues associated with prey-occupied plants. These characteristics are consistent with their recurrent presence in *O. yothersi* colonies in Chilean avocado orchards and with previous evidence of their attraction to mite-infested avocado plants and selected synthetic volatiles [5,7,9,40].

Plant-derived volatile cues can support conservation biological control by increasing the likelihood that natural enemies locate herbivore-infested patches [14,18,41,42]. Field attraction to methyl salicylate and green leaf volatiles has been demonstrated for other acarophagous coccinellids, including *Stethorus punctum picipes* [41,42,43,44]. The response of both predators to volatile blends associated with mite-infested avocado shoots therefore supports the possibility that plant cues help them locate *O. yothersi* colonies. Nevertheless, the expression of this behaviour under orchard conditions will also depend on volatile dispersion, background odours, prey density, plant condition and predator experience [14,18,45].

The next step should be to determine how these demographic and behavioural traits translate into prey consumption and pest suppression. Functional-response and prey-stage preference experiments would help establish the densities and developmental stages of *O. yothersi* most effectively exploited by each predator, while semi-field and field studies could evaluate their arrival, persistence and reproductive establishment on infested trees [2,11]. For *O. pygmaea*, the association of pupation with protected, humid microsites further suggests that litter availability and orchard-floor management may influence population persistence [8,9]. These complementary evaluations will clarify whether *P. histrio* and *O. pygmaea* can perform distinct or complementary functions within conservation and augmentative biological control programmes for the avocado red mite.

## 5. Conclusions

*Oligonychus yothersi* was a suitable prey for the development and reproduction of *P. histrio* and *O. pygmaea*, although both predators exhibited slower population growth than the mite. Their positive responses to volatile extracts from infested avocado shoots indicate that plant-associated olfactory cues may help predators locate prey habitat. In contrast, differences in their responses to prey-derived odours suggest species-specific searching strategies. These complementary demographic and behavioural traits support the continued evaluation of both beetles as natural enemies of the avocado red mite. However, their contribution to biological control must be confirmed through measurements of prey consumption, predator establishment and pest suppression under semi-field and orchard conditions.

## Figures and Tables

**Figure 1:**
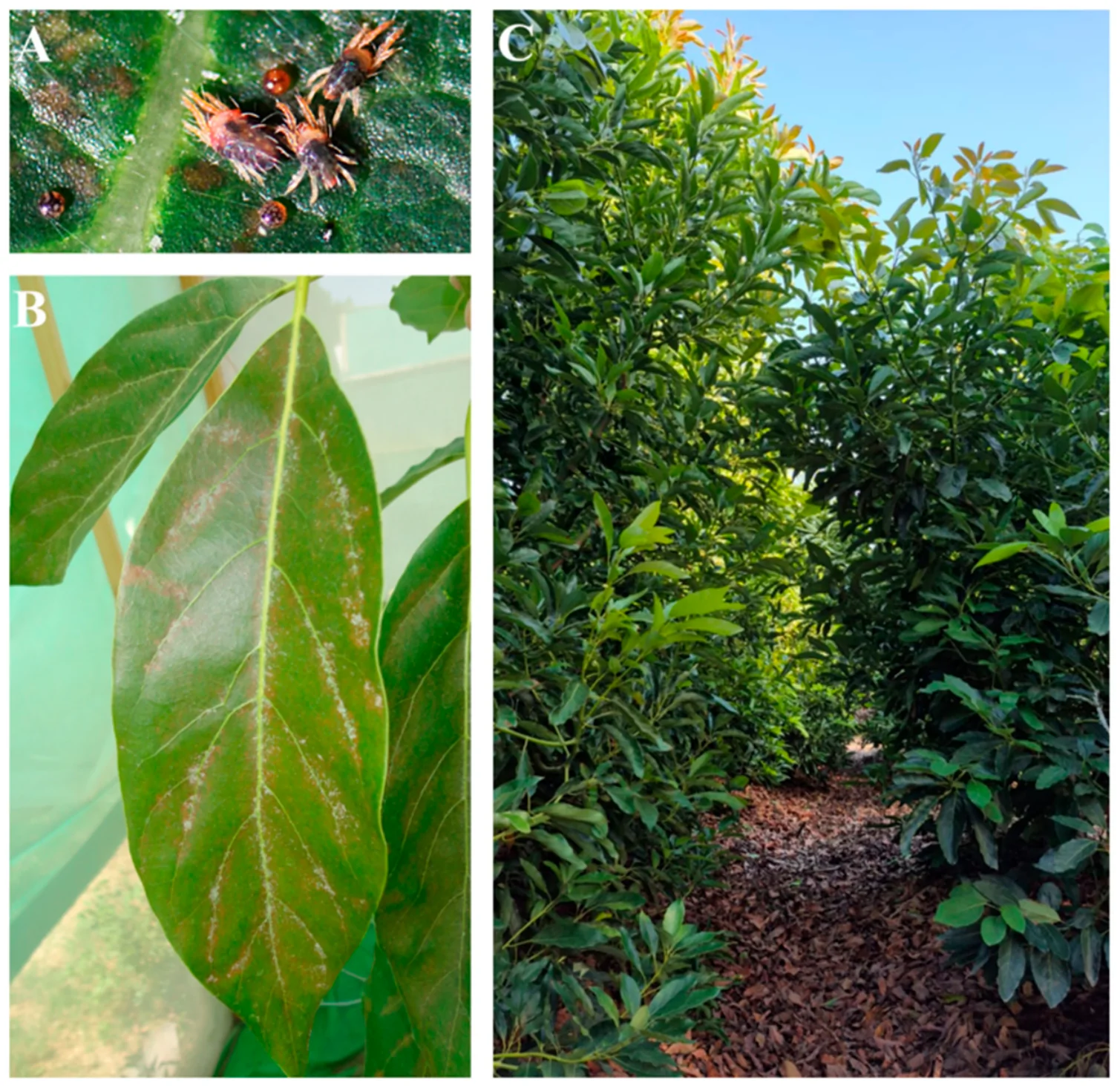
The avocado red mite is associated with foliar damage and orchard habitat in La Cruz, Chile. (**A**) Adult female, two adult males and eggs of *Oligonychus yothersi*. (**B**) Feeding damage on ‘Hass’ avocado leaves, characterised by reddish discolouration along the midrib and secondary veins on the adaxial surface, together with abundant mite exuviae. (**C**) Avocado orchard with leaf litter covering the soil surface and root zone, potentially providing protected and humid microsites for pupation of *Oligota pygmaea*.

**Figure 2:**
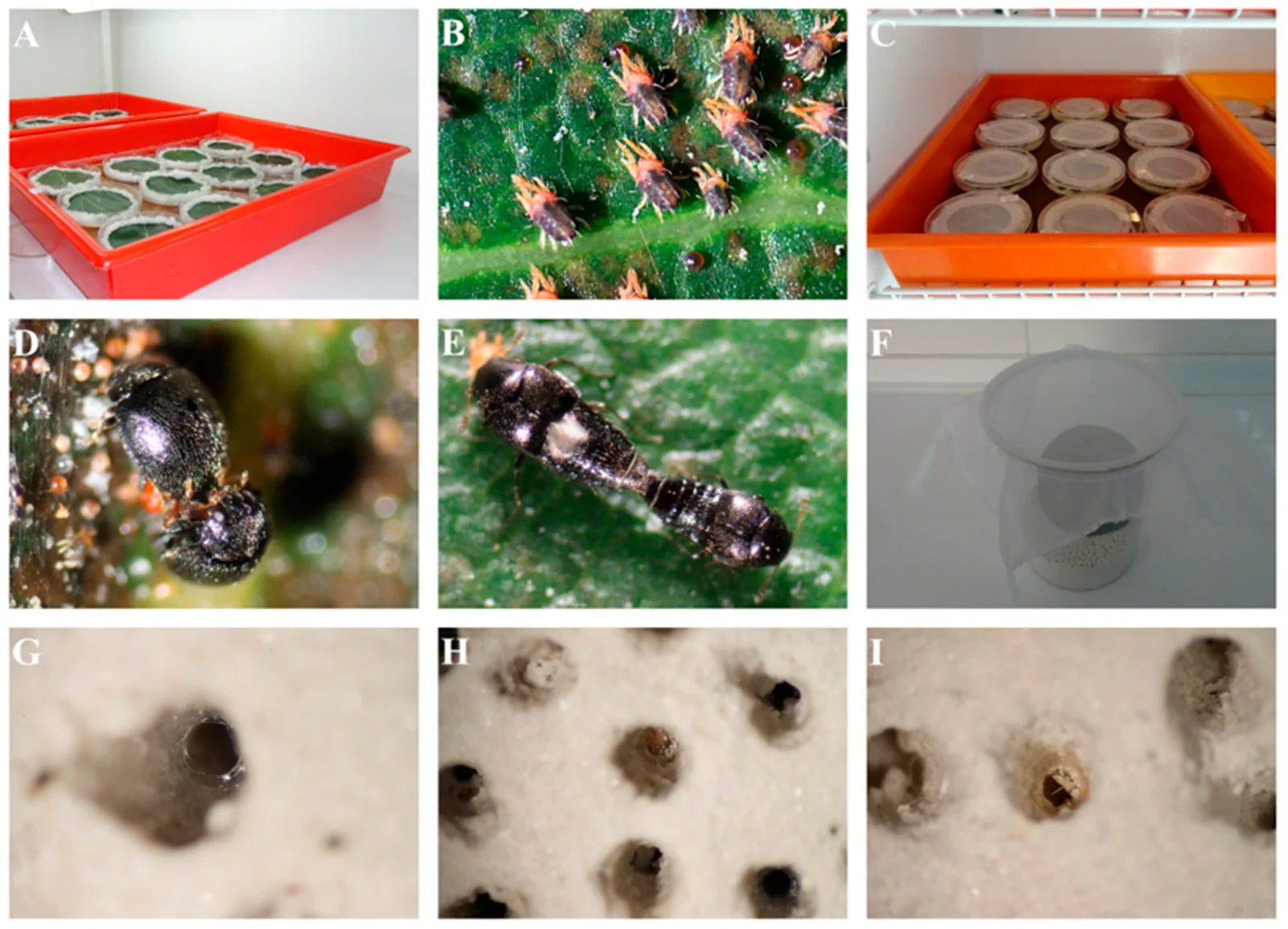
Rearing systems for the avocado red mite and its predatory beetles. (**A**) ‘Hass’ avocado leaf discs used for rearing *Oligonychus yothersi*. Each disc was placed on moist cotton inside a plastic Petri dish with a muslin-covered opening in its base. The dishes were maintained on damp sponges inside plastic trays to prevent leaf desiccation. (**B**) Initial *O. yothersi* colony on an avocado leaf disc. (**C**) Rearing unit for *Parastethorus histrio* and *Oligota pygmaea*, consisting of an infested avocado leaf disc enclosed between Petri dishes with a muslin-covered opening to prevent adult escape and excessive moisture accumulation. (**D**) Mating pair of *P. histrio*. (**E**) Mating pair of *O. pygmaea*. (**F**) Rearing container with a gypsum plaster substrate containing irregular cavities used by *O. pygmaea* for pupation under dark conditions. (**G**) Third-instar larva of *O. pygmaea* inside a selected cavity, beginning to seal the entrance before pupation. (**H**) Sealed pupation cavity containing an orange-coloured *O. pygmaea* pupa. (**I**) A newly emerged adult opening the sealed cavity with its mandibles.

**Figure 3:**
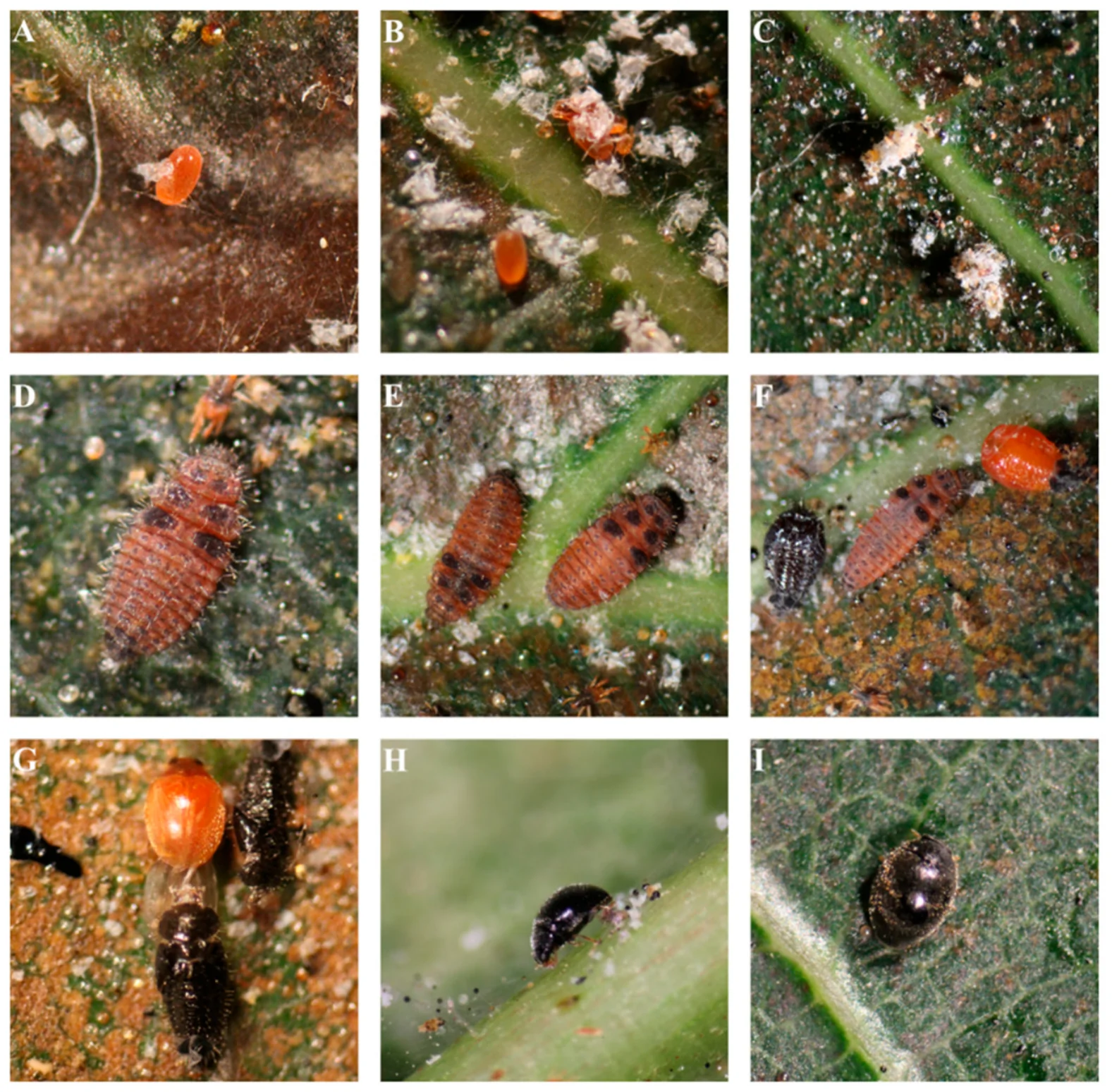
Developmental stages of *Parastethorus histrio* feeding on colonies of the avocado red mite, *Oligonychus yothersi*. (**A**) Egg. (**B**) Egg partially covered with mite exuviae. (**C**) Eggs completely covered with exuviae and dead bodies of *O. yothersi*. (**D**,**E**) Fourth-instar larvae. (**F**) Larva, newly formed deep-orange pupa and mature black pupa. (**G**) Newly emerged orange-coloured adults. (**H**) Lateral and (**I**) dorsal views of an adult.

**Figure 4:**
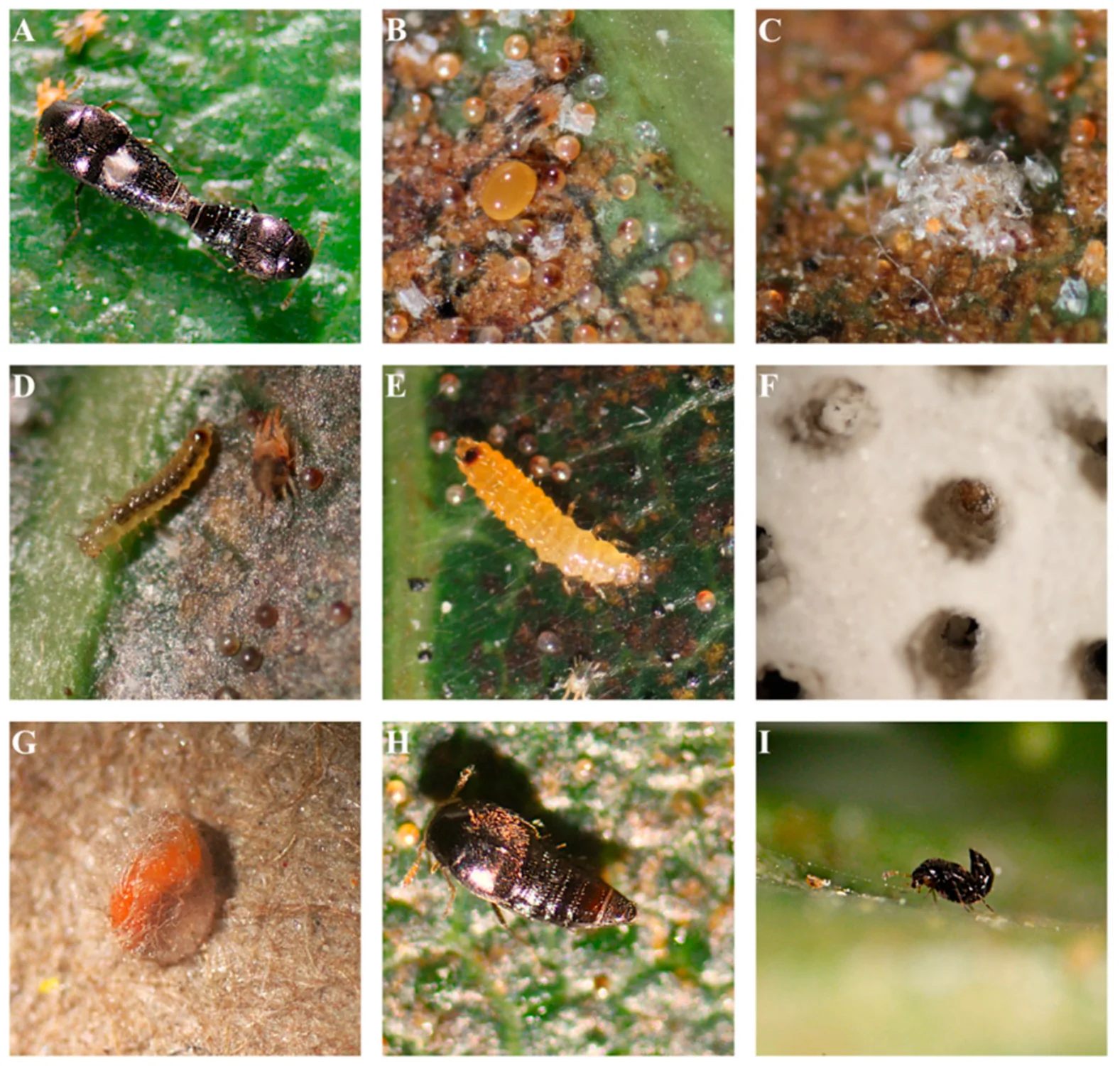
Developmental stages of *Oligota pygmaea* feeding on colonies of the avocado red mite, *Oligonychus yothersi*. (**A**) Mating pair. (**B**) Egg. (**C**) Egg covered with exuviae and dead bodies of *O. yothersi*. (**D**) Second-instar larva. (**E**) Late third-instar larva approaching pupation. (**F**) Pupation inside a cavity in the gypsum plaster substrate at the bottom of the rearing container. (**G**) Orange-coloured pupa removed from the pupation cavity for imaging. (**H**) Dorsal and (**I**) lateral views of an adult.

**Figure 5:**
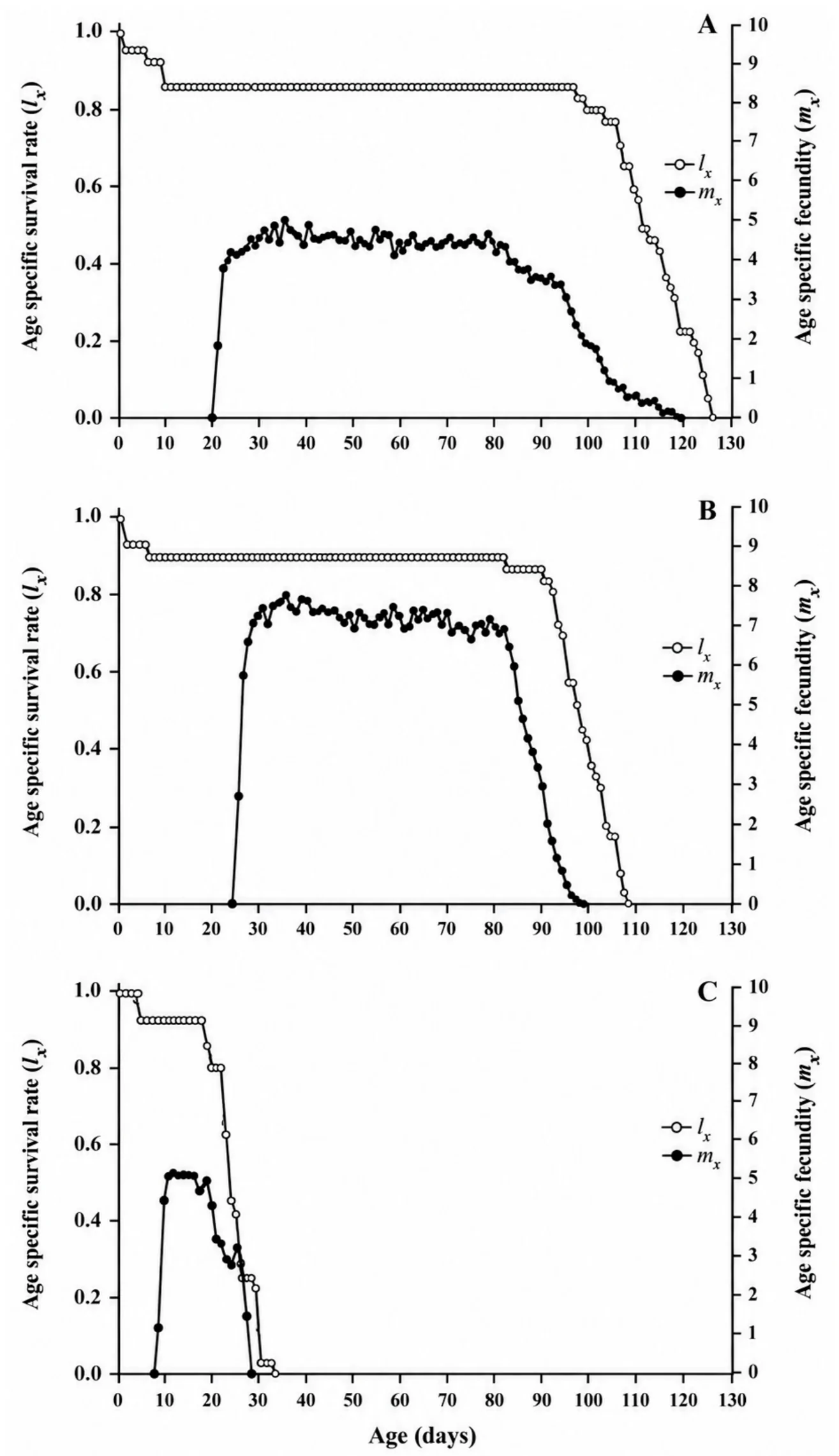
Age-specific survival (*l_x_*) and fecundity (*m_x_*) of the predatory beetles *Parastethorus histrio* and *Oligota pygmaea* and their prey, *Oligonychus yothersi*. (**A**) *P. histrio* females maintained on *O. yothersi* colonies on ‘Hass’ avocado leaf discs. (**B**) *O. pygmaea* females maintained under the same feeding conditions. (**C**) *O. yothersi* females maintained on ‘Hass’ avocado leaf discs. All cohorts were maintained at 25 ± 0.7 °C and 60 ± 5% RH. Age-specific survival rate (*l_x_*) represents the proportion of the initial cohort surviving to age *x*. In contrast, age-specific fecundity (*m_x_*) represents the mean number of eggs produced per female at age *x* per day.

**Figure 6:**
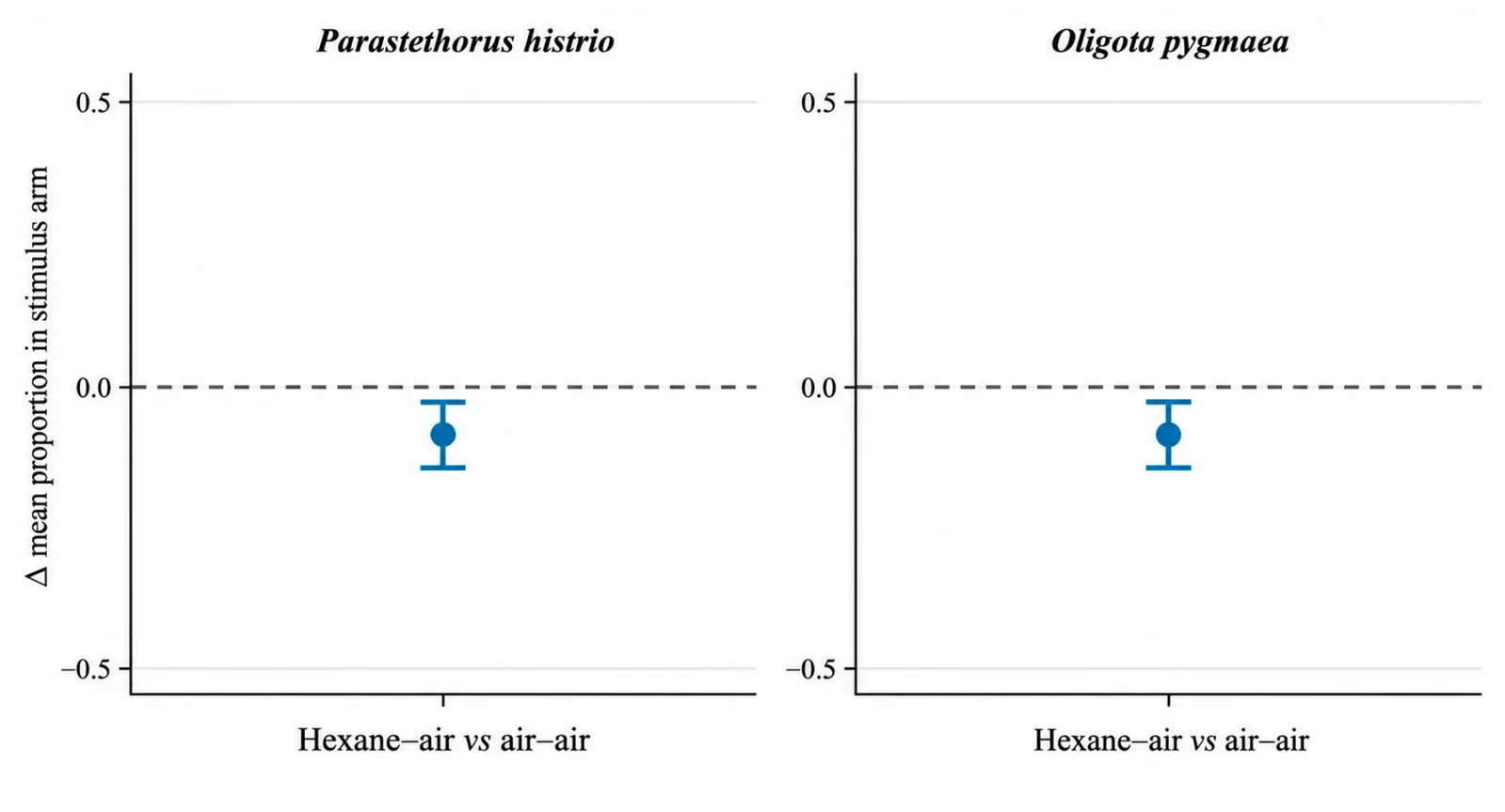
Assessment of solvent effects in two-choice olfactometer assays. Difference in the mean proportion of total trial time allocated to the stimulus arm (Δ) between the hexane–air and air–air conditions for *Parastethorus histrio* and *Oligota pygmaea*. Each trial lasted 600 s. Points represent the observed differences between conditions, and error bars indicate the corresponding 95% confidence intervals obtained by non-parametric bootstrapping with 10,000 resamples. The dashed horizontal line at Δ = 0 indicates no difference between the two conditions. Positive Δ values indicate greater stimulus-arm occupancy under the hexane–air condition relative to the air–air condition, whereas negative values indicate lower occupancy.

**Figure 7:**
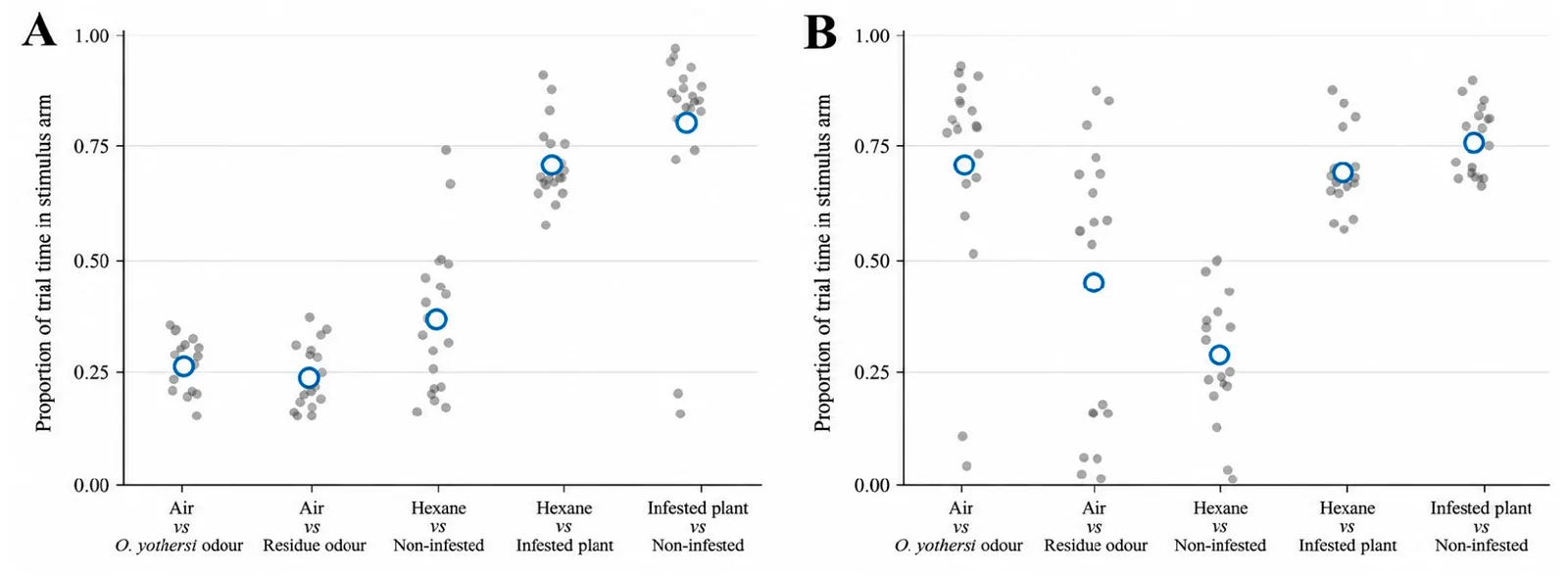
Olfactory responses of *Parastethorus histrio* and *Oligota pygmaea* in two-choice assays. Proportion of the total trial duration (600 s) spent in the stimulus arm by (**A**) *P. histrio* and (**B**) *O. pygmaea*. The comparisons were *Oligonychus yothersi* odour versus air, prey-associated residue odour versus air, non-infested avocado shoot extract versus hexane, mite-infested avocado shoot extract versus hexane, and mite-infested versus non-infested shoot extract. Grey points represent individual females, and coloured circles represent mean stimulus-arm occupancy for each comparison. Time spent in the stimulus, control and decision zones was expressed as a proportion of the total trial duration, with the three proportions summing to one. We evaluated pairwise contrasts using non-parametric bootstrapping with 10,000 resamples. We calculated differences in mean proportions (Δ) and their 95% bootstrap confidence intervals separately for each zone. Air–air assays were used as the reference for prey-derived odours and the direct comparison between mite-infested and non-infested shoot extracts, whereas hexane–air assays were used as the reference for comparisons involving plant extracts and the solvent vehicle. Preliminary air–air and hexane–air assays characterised basal spatial allocation and the solvent effect, respectively.

**Table 1:** Embryonic and post-embryonic development, and female survival of *Parastethorus histrio* and *Oligota pygmaea* feeding on colonies comprising all developmental stages of *Oligonychus yothersi* on ‘Hass’ avocado leaf discs at 25 ± 0.7 °C and 60 ± 5% RH. Developmental duration is presented as mean ± SD, whereas survival is expressed as a percentage (initial n = 30 individuals per predator species).

Stage	*Parastethorus histrio*	*Oligota pygmaea*	*Parastethorus histrio*	*Oligota pygmaea*
	Survival (%)		Development (d)	
Egg	96.6	93.3	5.1 ± 0.4 a	4.0 ± 0.7 b
Larvae:				
First instar	93.3	93.3	2.2 ± 0.6 a	1.9 ± 0.5 a
Second instar	93.3	90	2.4 ± 0.5 a	1.9 ± 0.7 a
Third instar	86.6	90	2.3 ± 0.5 b	3.7 ± 0.7 a
Fourth instar	86.6	-	3.2 ± 0.6	-
Pupae	86.6	90	4.5 ± 0.5 b	13.0 ± 1.1 a
Egg to adult	86.6	90	19.6 ± 1.2 b	24.4 ± 1.7 a

Note: Means (n = 30) sharing a letter do not differ significantly according to Student’s test (*p* < 0.05).

**Table 2:** Embryonic and post-embryonic development, and female survival of *Oligonychus yothersi* on ‘Hass’ avocado leaf discs at 25 ± 0.7 °C and 60 ± 5% RH. Developmental duration is presented as mean ± SD, whereas survival is expressed as a percentage (n = 30).

Stage	*Oligonychus yothersi*	
	Survival (%)	Development (d)
Egg	100	4.2 ± 0.4
Larva	93.3	1.8 ± 0.4
Protonymph	93.3	1.0 ± 0.2
Deutonymph	93.3	1.8 ± 0.4
Egg to adult	93.3	8.8 ± 0.4

**Table 3:** Reproductive parameters of the predatory beetles *Parastethorus histrio* and *Oligota pygmaea* and their prey, *Oligonychus yothersi*, under controlled conditions. Predators were provided with colonies containing all developmental stages of *O. yothersi* on ‘Hass’ avocado leaf discs. Values are presented as mean ± SD at 25 ± 0.7 °C and 60 ± 5% RH.

Parameter	Predators		Prey
	*Parastethorus histrio*	*Oligota pygmaea*	*Oligonychus yothersi*
Pre-oviposition period (d)	3.5 ± 0.50 a	2.4 ± 0.5 b	1.1 ± 0.4 c
Oviposition period (d)	79.5 ± 8.5 a	62.5 ± 5.5 b	15.0 ± 3.2 c
Post-oviposition period (d)	14.5 ± 3.7 a	10.9 ± 3.2 a	1.2 ± 1.2 b
Total eggs female^−1^	351.8 ± 41.1 b	441.6 ± 51.5 a	68.1 ± 16.1 c
Mean eggs female^−1^ d^−1^	4.4 ± 0.2 b	7.0 ± 0.4 a	4.6 ± 0.6 b
Longevity (d)	97.4 ± 8.2 a	76.0 ± 6.06 b	17.2 ± 3.6 c
Sex ratio (Female:Male)	1:0.86	1:0.79	1:0.31

Note: Means (n = 30) sharing a letter do not differ significantly according to Tukey’s multiple comparisons test (*p* < 0.05).

**Table 4:** Fertility life-table parameters of the predatory beetles *Parastethorus histrio* and *Oligota pygmaea* and their prey, *Oligonychus yothersi*, under controlled conditions. Predators were provided with *O. yothersi* colonies on ‘Hass’ avocado leaf discs, whereas *O. yothersi* was maintained on non-infested leaf discs. Values are presented as mean ± SE at 25 ± 0.7 °C and 60 ± 5% RH.

Parameter	*Parastethorus histrio*	*Oligota pygmaea*	*Oligonychus yothersi*
Net reproductive rate (*R*_0_)	160.350 ± 18.744 b	218.625 ± 25.485 a	47.523 ± 11.206 c
Intrinsic rate of increase (*r_m_*)	0.126 ± 0.006 b	0.129 ± 0.003 b	0.245 ± 0.016 a
Finite rate of increase (*λ*)	1.135 ± 0.007 b	1.138 ± 0.004 b	1.277 ± 0.021 a
Mean generation time (*T*)	40.059 ± 2.963 b	41.541 ± 1.506 a	15.663 ± 0.974 c
Doubling time (*DT*)	5.468 ± 0.292 a	5.344 ± 0.158 a	2.811 ± 0.160 b

Note: Means (n = 30) sharing a letter do not differ significantly according to Tukey’s multiple comparisons test (*p* < 0.05).

**Table 5:** Semiquantitative abundance of volatile organic compounds detected in extracts from non-infested and *Oligonychus yothersi*-infested ‘Hass’ avocado shoots under semi-field conditions during early autumn. Values are expressed as methyl jasmonate equivalents (µg mL^−1^) and presented as mean ± SE (n = 6 plants per treatment).

Compounds *	Non-Infested	Infested
***Green Leaf Volatile***		
3-Hexanone	1.18 ± 0.24	0.89 ± 0.12
2-Hexanone	1.35 ± 0.29	0.98 ± 0.06
3-Hexanol (Hexan-3-ol)	1.27 ± 0.27	0.99 ± 0.13
***Alkanes***		
2,4-Dimethylheptane	1.99 ± 0.27	1.44 ± 0.28
4,5-Dimethyloctane	2.93 ± 0.30	2.46 ± 0.10
4,7-Dimethylundecane	5.54 ± 0.58	-
***Terpenoids***		
(1R,5R)-2,6,6-Trimethylbicyclo[3.1.1]hept-2-ene (α-Pinene)	5.48 ± 1.12 **	1.72 ± 0.13
6,6-Dimethyl-2-methylenebicyclo[3.1.1]heptane (β-Pinene)	2.31 ± 0.44	1.55 ± 0.15
7-Methyl-3-methylene-1,6-octadiene (Myrcene)	-	1.35 ± 0.11
3,7,7-Trimethylbicyclo[4.1.0]hept-3-ene (3-Carene)	2.91 ± 0.49	-
(4R)-4-Isopropenyl-1-methylcyclohexene ((R)-(+)-Limonene)	3.76 ± 0.77	108.05 ± 5.16 **
3,7-Dimethyl-1,3,6-octatriene (β-Ocimene)	-	1.52 ± 0.39
3,7,11-Trimethyl-1,3,6,10-dodecatetraene (α-Farnesene)	-	1.37 ± 0.28
(2E)-3,7-Dimethyl-2,6-octadien-1-ol (Geraniol)	-	2.52 ± 0.16
***Ester***		
methyl 2-hydroxybenzoate (Methyl salicylate)	-	0.50 ± 0.07

Note: * IUPAC name (Common name). ** Significant differences between non-infested and *O. yothersi*-infested plants, as determined by Student’s *t*-test (*p* < 0.05). (-) Compounds not detected by GC-MS.

## Data Availability

The original contributions arising from this research are presented in the manuscript. Further information may be obtained from the corresponding authors upon request.

## Supplementary Materials

The following supporting information can be downloaded at: https://www.mdpi.com/article/10.3390/insects17101048/s1, Supplementary Data S1: Raw data from the olfactory responses of *Parastethorus histrio* and *Oligota pygmaea* to volatiles from infested avocado shoots and prey-associated odours.
